# Dual inhibition of SGLT 1 and 2 by sotagliflozin alleviates intracellular sodium overload and improves cardiometabolic remodelling in uraemic cardiomyopathy

**DOI:** 10.1093/cvr/cvag167

**Published:** 2026-08-11

**Authors:** Megan Young, Anja Karlstaedt, Sanushi Dambure, Jack J J J Miller, Tong Guo, Xin Zhou, Zenouska Ramchunder, Jair Gonzalez Marques Junior, Muriel Nobles, Andrew Tinker, Kyuongmin Kim, Amanda R Purcell, Sarah J Glastras, Fenn Cullen, Thomas R Eykyn, Alex Von Kriegsheim, Blanca Rodriguez, Michelangelo Campanella, Michael J Shattock, Nay Aung, Claudia P Cabrera, Magdi M Yaqoob, Dunja Aksentijevic

**Affiliations:** William Harvey Research Institute, John Vane Science Centre, Barts and the London Faculty of Medicine and Dentistry, Queen Mary University of London, Charterhouse Square, London EC1M 6BQ, UK; Department of Cardiology, Smidt Heart Institute, Cedars Sinai Medical Center, Los Angeles, USA; William Harvey Research Institute, John Vane Science Centre, Barts and the London Faculty of Medicine and Dentistry, Queen Mary University of London, Charterhouse Square, London EC1M 6BQ, UK; The MR Research Centre and The PET Centre, Clinical Medicine, Aarhus University, Aarhus 8200, Denmark; Clarendon Laboratory, Department of Physics, University of Oxford, Oxford, UK; William Harvey Research Institute, John Vane Science Centre, Barts and the London Faculty of Medicine and Dentistry, Queen Mary University of London, Charterhouse Square, London EC1M 6BQ, UK; Department of Computer Science, University of Oxford, Oxford, UK; William Harvey Research Institute, John Vane Science Centre, Barts and the London Faculty of Medicine and Dentistry, Queen Mary University of London, Charterhouse Square, London EC1M 6BQ, UK; Cancer Research UK Scotland Centre, Institute of Genetics and Cancer, University of Edinburgh, Edinburgh, UK; William Harvey Research Institute, John Vane Science Centre, Barts and the London Faculty of Medicine and Dentistry, Queen Mary University of London, Charterhouse Square, London EC1M 6BQ, UK; William Harvey Research Institute, John Vane Science Centre, Barts and the London Faculty of Medicine and Dentistry, Queen Mary University of London, Charterhouse Square, London EC1M 6BQ, UK; Department of Cardiology, Smidt Heart Institute, Cedars Sinai Medical Center, Los Angeles, USA; University of Sydney, Faculty of Medicine and Health, Sydney, NSW, Australia; University of Sydney, Faculty of Medicine and Health, Sydney, NSW, Australia; William Harvey Research Institute, John Vane Science Centre, Barts and the London Faculty of Medicine and Dentistry, Queen Mary University of London, Charterhouse Square, London EC1M 6BQ, UK; School of Biomedical Engineering and Imaging Sciences, King’s College London, St Thomas’ Hospital, London, UK; British Heart Foundation Centre of Research Excellence, King’s College London, London, UK; Cancer Research UK Scotland Centre, Institute of Genetics and Cancer, University of Edinburgh, Edinburgh, UK; Department of Computer Science, University of Oxford, Oxford, UK; William Harvey Research Institute, John Vane Science Centre, Barts and the London Faculty of Medicine and Dentistry, Queen Mary University of London, Charterhouse Square, London EC1M 6BQ, UK; UMR 9018 CNRS, Institute Gustave Roussy, Villejeuif, France; Department of Biomedical Sciences, University of Padua, Padua, Italy; British Heart Foundation Centre of Research Excellence, King’s College London, London, UK; Rayne Institute, School of Cardiovascular and Metabolic Medicine and Sciences, Faculty of Life Sciences & Medicine, King’s College London, St Thomas’ Hospital, London, UK; William Harvey Research Institute, John Vane Science Centre, Barts and the London Faculty of Medicine and Dentistry, Queen Mary University of London, Charterhouse Square, London EC1M 6BQ, UK; William Harvey Research Institute, John Vane Science Centre, Barts and the London Faculty of Medicine and Dentistry, Queen Mary University of London, Charterhouse Square, London EC1M 6BQ, UK; National Institute for Health and Care Research, Barts Cardiovascular Biomedical Research Centre, Queen Mary University of London, London, UK; William Harvey Research Institute, John Vane Science Centre, Barts and the London Faculty of Medicine and Dentistry, Queen Mary University of London, Charterhouse Square, London EC1M 6BQ, UK; William Harvey Research Institute, John Vane Science Centre, Barts and the London Faculty of Medicine and Dentistry, Queen Mary University of London, Charterhouse Square, London EC1M 6BQ, UK

**Keywords:** CKD, Uraemic cardiomyopathy, Intracellular sodium, Cardiac metabolism, SGL T1/2 inhibition, Sotagliflozin

## Abstract

**Aims:**

Chronic kidney disease (CKD) is associated with uraemic cardiomyopathy characterized by early metabolic dysfunction. Elevated myocardial intracellular sodium (Naᵢ) has emerged as a driver of cardiometabolic remodelling, however, its role in CKD and therapeutic modulation remains unclear. We investigated whether dual sodium-glucose cotransporter (SGLT)1/2 inhibition with sotagliflozin (SOTA) targets Naᵢ and improves cardiac metabolism in CKD.

**Methods and results:**

CKD was induced in male Wistar rats by 5/6 nephrectomy and assessed after 4 weeks. Cardiac phenotype was evaluated using *in vivo* echocardiography and *ex vivo* Langendorff perfusion combined with ^23^Na and ^31^P NMR spectroscopy. CKD hearts exhibited preserved systolic but impaired diastolic function and a marked elevation in myocardial Naᵢ, identifying Naᵢ overload as an early feature of uraemic cardiomyopathy. Cardiac metabolomic profiling and flux modelling demonstrated widespread suppression of central carbon metabolism, redox imbalance despite preserved PCr/ATP. *In silico* electrophysiological simulations predicted Naᵢ-driven Ca^2+^ dysregulation consistent with *in vivo* diastolic dysfunction. Chronic SOTA treatment (3 weeks, *in vivo*) normalized myocardial Naᵢ and partially reversed metabolic remodelling. Acute SOTA exposure (20 min, Langendorff-perfusion) similarly reduced Naᵢ in CKD hearts but not in controls, indicating a direct, disease-selective myocardial effect. Naᵢ normalization was accompanied by improved redox state and restoration of mitochondrial metabolic flux, without changes in expression of canonical Na ^+^ -handling proteins.

**Conclusion:**

Myocardial Naᵢ overload emerges as an early and potentially modifiable feature of uraemic cardiomyopathy. Dual SGLT1/2 inhibition with SOTA directly lowers Naᵢ and improves cardiac metabolic homeostasis, supporting Naᵢ as a mechanistically relevant and potentially targetable pathway in CKD-related cardiac remodelling.

## Graphical Abstract

**Graphical Abstract:**
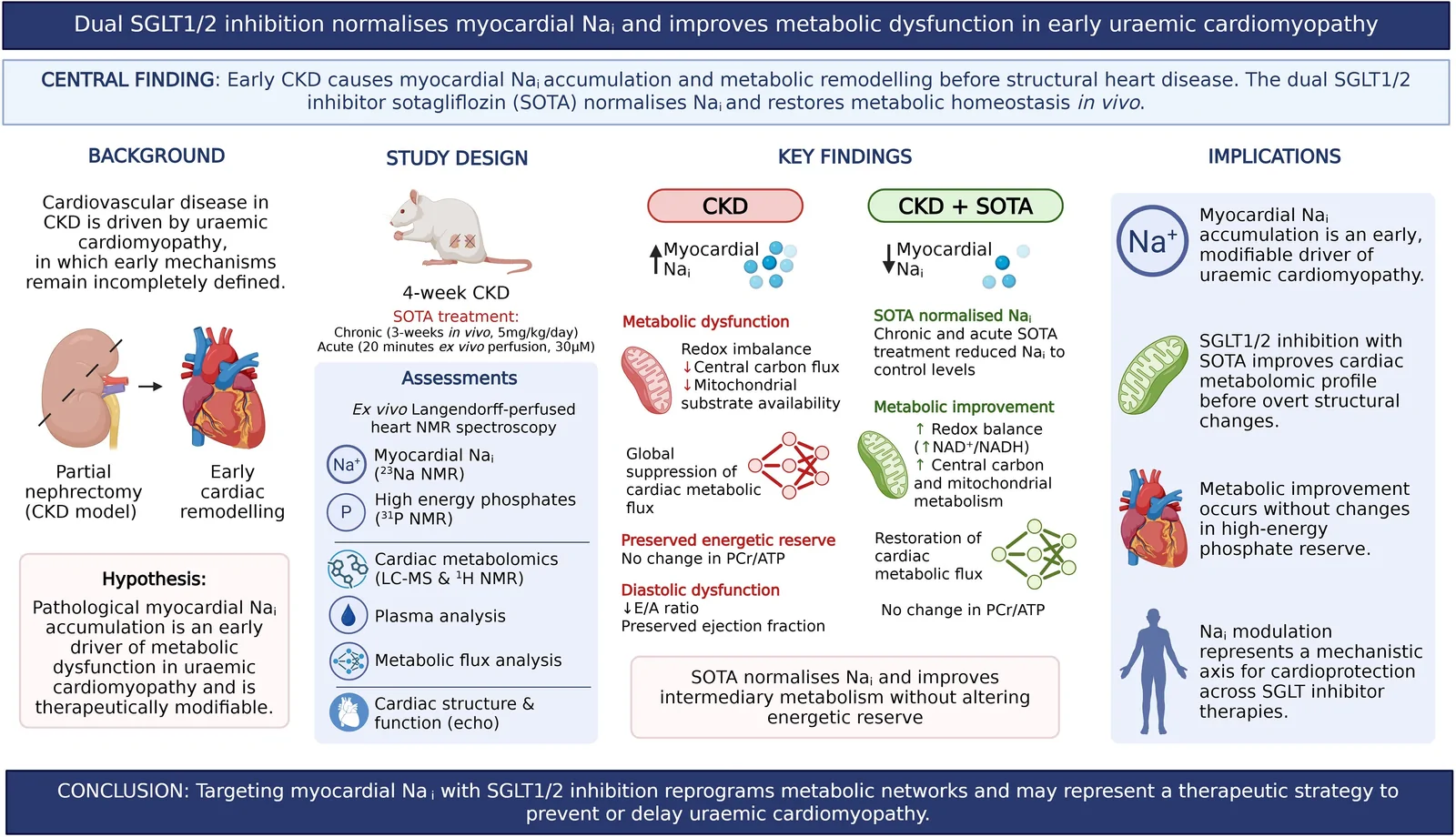

**Time of primary review: 38 days**

## 1. Introduction

Chronic kidney disease (CKD) affects 14% of the global population, and cardiovascular disease represents a leading cause of mortality among affected individuals.^1^ CKD-driven cardiomyopathy (uraemic cardiomyopathy) is characterized by heart failure (HF), with both preserved and reduced ejection fraction (HFpEF, HfrEF), myocardial fibrosis, hypertrophy, and arrhythmia.^2^ A myriad of driving factors contribute to the aetiology of pathological cardiac remodelling in CKD, including hypertension, anaemia, diabetes, inflammation, and oxidative stress.^2,3^

Myocardial intracellular Na (Na_i_) overload is a common feature of HF irrespective of species, aetiology, and duration.^4,5^ Increased Na^+^ influx and enhanced late sodium current (late INa) have been demonstrated in atrial biopsies from patients with HFpEF^6^ and in murine HFpEF models.^7^ Elevated myocardial Na_i_ has been shown to decrease Gibbs free energy of ATP hydrolysis and alter mitochondrial electron transport chain function, effects that can be reversed by lowering Na_i_.^8^ Furthermore, increased Na_i_ precedes and drives cardiac metabolic remodelling, triggering a shift in substrate utilization prior to functional deterioration and energetic compromise.^9^

CKD is known to cause disruption in systemic ion homeostasis, including Na_i_,^10–13^ with tissue-level Na_i_ accumulation (e.g. skin and muscle) reported in CKD patients.^14^ The possibility that elevated Na_i_ drives cardiac dysfunction and metabolic remodelling in uraemic cardiomyopathy aligns with commonly observed diastolic Ca^2+^ overload, contractile dysfunction, arrhythmogenesis, and left ventricular hypertrophy (LVH).

However, despite strong indirect evidence, no studies to date have measured Na_i_ in intact beating hearts from CKD animals, nor directly examined its contribution to cardiac dysfunction in CKD. As myocardial Na_i_ is a dynamic and reversible modulator of mitochondrial metabolism,^8,9^ targeting Na_i_ represents an attractive and novel therapeutic strategy to correct metabolic derangements in CKD-associated cardiomyopathy.

Sotagliflozin (SOTA) is a dual sodium-glucose cotransporter (SGLT1/2) inhibitor that targets renal SGLT2 and intestinal SGLT1 and is used clinically for glycaemic control in type 2 diabetes, and to prevent progression of albuminuric-CKD and/or HF.^15,16^ Following the SOLOIST-WHF and SCORED trials, SOTA became the first dual SGLT1/2 inhibitor approved for HF across the spectrum of ejection fraction, including patients with CKD.^17^ In addition to reducing cardiovascular mortality and hospitalization, SOTA improves cardiorenal outcomes in CKD patients.^17,18^ While SGLT2 expression is minimal in the heart, SGLT1 is expressed in human cardiomyocytes and contributes to myocardial glucose uptake alongside GLUT1 and GLUT4.^16,19,20^ In pathological states such as obesity and type 2 diabetes, SGLT1 has been associated with increased Na_i_ and glucose influx into cardiomyocytes.^21^

Despite these advances, the cellular mechanisms underlying the cardioprotective effects of SGLT inhibition remain incompletely understood.^22–26^ SGLT2 inhibitors, including empagliflozin, dapagliflozin, and canagliflozin, have been shown to modulate [Na_i_] and attenuate pathologically enhanced late INa in isolated cardiomyocytes independently of SGLT2^22,27^ suggesting off-target or systemic effects. These findings have been reported across models of HFrEF,^28^ HFpEF,^6,7^ and diabetes,^21^ although most studies to date have been limited to isolated cardiomyocytes. Whether dual SGLT1/2 inhibition can modulate myocardial Na_i_ and thereby improve cardiac energetics and mechano-energetic coupling in uraemic cardiomyopathy remains unknown.

Here, we used an integrated *in vivo*, *ex vivo,* and *in silico* approach, including *in situ* multiple quantum-filtered ^23^Na and ^31^P NMR spectroscopy, to investigate myocardial Na_i_, energetics, and the therapeutic potential of SOTA in a partial nephrectomy (PN)-induced model of CKD. This study is the first to quantify Na_i_ in intact beating CKD hearts and to evaluate the effects of dual SGLT1/2 inhibition on cardiac Na_i_ homeostasis and metabolism in this context.

## 2. Methods

### 2.1 Animals

For all experiments in this study, 300 g male Wistar rats (study total n = 57) were purchased from Charles River Laboratories (UK). Animals were housed in individually ventilated cages and maintained under controlled temperature (22 ± 2°C) on a 12:12 h light and dark cycle with access to standard chow diet (irradiated Labdiet 5LF2 – EURodent Diet 14%) and water *ad libitum.* Enrichment was provided in the form of tunnels and chew sticks. All animal experiments were in keeping with the UK Home Office Animals (Scientific Procedures) Act, 1986. At the end of the study, all animals were terminally anaesthetized by intraperitoneal injection of pentobarbital (150 mg/kg).

### 2.2 Experimental CKD model

CKD was induced using an established two-stage PN surgical procedure.^3,29–34^ Experiments were performed 4-weeks after the second surgery. Controls were sham operated by removing the renal capsule and replacing the intact kidney. Blood pressure was not measured as hypertension has already been well characterized in this PN CKD model,^3,29–34^ and additional procedures were avoided in accordance with the UK Home Office 3Rs principles.

### 2.3 In vivo drug administration

PN CKD rats (*n* = 12) were randomized to receive SOTA (MedChem Express, UK) at 5 mg/kg/day given in drinking water (drug dissolved in DMSO; final DMSO concentration 0.05% v/v). Drug treatment was initiated 1 week after the second stage surgery and given for the remaining 3 weeks of the study (chronic exposure). Dose selection (5 mg/kg/day) was informed by prior pilot experiments (48 h dosing) demonstrating biological activity without adverse effects, and SOTA was administered *via* drinking water for continuous exposure. Consistent dosing was achieved through monitoring both body weight and water consumption of the animals before and during drug treatment. Water consumption was unchanged by addition of the drug.

### 2.4 Transthoracic echocardiography

For the assessment of cardiac function, echocardiography was performed as previously described.^35^ Anaesthesia was induced with 5% isoflurane and maintained at 2% isoflurane throughout imaging. Echocardiography images were recorded using a Vevo-3100 imaging system with a MX201 transducer (VisualSonics). Ejection fraction was calculated from M-mode measurements in the parasternal short axis view. Diastolic function was assessed using pulse wave doppler in four-chamber view.

### 2.5 In situ ^23^Na and ^31^P NMR Langendorff perfusion protocols

Upon termination, hearts were rapidly excised and cannulated on an NMR-compatible Langendorff perfusion rig. Hearts were perfused with phosphate-free Krebs-Henseleit (KH) buffer and subsequently lowered into the centre of the magnet for real-time multiple quantum filtered ^23^Na NMR (triple quantum filtered; TQF) quantification of [Na]_i_ and ^31^P NMR assessment of cardiac energetics as previously described.^4,36^

All in situ NMR Langendorff rat heart perfusion experiments were carried out on a Bruker Avance III 9.4 T 400 MHz vertical wide-bore spectrometer (Bruker, Karlsruhe, Germany) equipped with triple-axis imaging gradients, a microimaging probe and 30 mm dual tune ^23^Na/^31^P coil. Fully relaxed ^31^P experiments were acquired with a 60° flip angle, 128 scans, a repetition time of 3.8 s and a total experiment duration of 8 min. ^31^P spectra were quantified via the OXSA implementation of AMARES as previously described to measure pH and assess cardiac energetics.^37–39^

TQF ^23^Na experiments were acquired with 192 scans, 2048 data points, sweep width of 50 ppm, an acquisition time of 194 ms, pre-scan delay of 100 ms and a total acquisition time of 59 s. The mixing time (τ_m_ = 3.6 ms) was calibrated for the maximum TQF signal. An exponential line broadening factor of 15 Hz was applied prior to Fourier transformation and subsequent baseline correction. Peak integrals were measured using Bruker Top spin version 4.3.0 software. Na_i_ quantification following TQF scans was performed as previously described.^4,36,40^

For chronic SOTA experiments, acquisitions were performed during a 20-min period with KH perfusion (Sham *n* = 7, CKD *n* = 6, CKD + chronic SOTA *n* = 5). For acute SOTA experiments, SOTA was resuspended in DMSO and made to a final concentration of 30 µM in KH buffer (0.02% (v/v) DMSO). Acquisitions were made following 20-min stabilization period with KH containing SOTA (n = 5, CKD + acute SOTA). An additional cohort of naïve Wistar rat hearts (n = 6) were perfused with KH for 20 min followed by 20-min perfusion with KH containing SOTA, acquisitions were made both pre- and post- drug treatment. Acute isolated heart experiments were conducted as proof-of-principle to exclude direct energetic or toxic cardiac effects of SOTA and do not reflect therapeutic plasma exposure. Baseline Na_i_ values were measured in Sham and Naïve hearts. Because these experiments were conducted on different days, data were normalized to the group mean for each day to account for technical variation. No biological differences were observed between Sham and Naive hearts; therefore, these groups were pooled as a single control group.

### 2.6 Cardiomyocyte isolation

A cohort of naïve Wistar rat hearts (*n* = 3) were perfused for cardiomyocyte isolation by collagenase digestion as previously described.^3^ Isolated cardiomyocytes were used to demonstrate cardiomyocyte-specific SGLT1 expression and to exclude the possibility that SGLT1 expression detected in whole-heart tissue arises solely from non-myocyte cell populations.

### 2.7 Biochemical analysis

Tissues (liver, skeletal muscle, heart, and kidney) were rapidly collected upon termination and snap frozen in liquid nitrogen.^41^ The frozen heart weight was normalized to tibia length to assess the degree of cardiac hypertrophy (heart weight:tibia length). Blood samples taken from the thoracic cavity at the time of sacrifice were centrifuged at 1500 x g to obtain plasma. All samples were stored at −80°C until biochemical analysis. Plasma samples were analysed by the Clinical Biochemistry Laboratory at Addenbrookes Hospital, Cambridge.

### 2.8 Metabolomic analysis

Snap frozen and pulverized tissue (liver, skeletal muscle (gastrocnemius and soleus), kidney, heart) were analysed using semi-targeted ^1^H NMR high-resolution spectroscopy as previously described.^41^ Cardiac tissue was also analysed by targeted LC/MS-MS analysis as previously described.^42^

### 2.9 qPCR

RNA was isolated from cardiac tissue (Cat:74704, Qiagen) and reverse transcribed (Cat:4374967, Applied Biosystems). For qPCR, gene specific primer sequences are shown in Supplementary material online, *Table S1*. qPCR reactions were performed in triplicate using SYBR Green (Cat:600892, Agilent) with the cycling conditions: 95°C for 3 min, 40 cycles of 95°C for 5 s and 60°C for 60 s on QuantStudio 5 (Applied Biosystems). The relative quantity of each gene was calculated using ΔΔCT method with 36B4 as endogenous control.

### 2.10 Western blotting

Total protein was extracted from cardiac tissue by homogenization in lysis buffer supplemented with protease and phosphatase inhibitors, followed by centrifugation. For NaKATPase analyses (FXYD1/PLM, phospho-PLM, ATP1A1, ATP1A2, SGLT1), samples were prepared in RIPA buffer and quantified using the Pierce Gold BCA assay (Thermo Scientific). Proteins (10 µg) were separated on 5% TGX gels and transferred to PVDF membranes. Total protein was visualized with Ponceau S. For translocator protein (TSPO) and thymopoietin (TMPO) analyses, samples were prepared in Tris-based lysis buffer (50 mM Tris, 150 mM NaCl, 1% Triton X-10) and quantified using the DC Protein Assay (Bio-Rad). Proteins (120 µg) were separated on 12.5% polyacrylamide gels and transferred to PVDF membranes. Total protein was visualized with No-Stain™ Protein Labelling Reagent (Invitrogen™, A44717). After blocking in 5% milk, membranes were incubated overnight at 4°C with primary antibodies: FXYD1/PLM (Abcam ab76597, 1:1000), phospho-PLM (S68; 0.5 µg/mL), ATP1A1 (Invitrogen MA1-16731, 1:1000), ATP1A2 (RayBiotech 144-60486-50, 1:1000), SGLT1 (Abcam ab14686, 1:1000), TMPO (Proteintech 14651-1-AP, 1:1000), and TSPO (Proteintech 68137-1-Ig, 1:1000). Appropriate HRP-conjugated secondary antibodies were applied for 1.5 h at room temperature: Cytiva NA934 (1:5000), Cytiva NA931 (1:5000), Invitrogen 31480 (1:5000), Agilent P044801-2 (1:4000), and Agilent P044701-2 (1:4000). Proteins were visualized using enhanced chemiluminescence (SuperSignal™ West Pico PLUS or Pierce™ ECL substrate, Thermo Scientific) and imaged using either an Azure 600 (Azure Biosystems) or ChemiDoc imaging system (Bio-Rad), depending on protocol. Densitometric analysis was performed using ImageJ or Image Lab software, with protein expression normalized to total protein. For TSPO and TMPO proteins, to account for potential inter-gel variability, all samples were normalized to a common control sample (pooled baseline myocardial tissue) that was included on every gel, ensuring that differences in exposure time or membrane development did not affect relative quantification.

### 2.11 RNA sequencing

Total RNA was extracted from snap-frozen cardiac tissue from CKD, controls, and chronic SOTA-treated groups (*n* = 5 per group) and used for library preparation. Poly(A)+ mRNA was enriched from total RNA using oligo(dT)-attached magnetic beads and fragmented prior to cDNA synthesis. First- and second-strand cDNA synthesis was performed using random N6 primers. Double-stranded cDNA fragments were end-repaired and 5′-phosphorylated, with the addition of a single adenine residue at the 3′ end, followed by ligation of bubble-shaped adapters with a complementary 3′-thymine overhang. Adapter-ligated fragments were PCR amplified using specific primers. PCR products were denatured, and single-stranded circular DNA libraries were generated using a bridged primer. Libraries passing quality control were sequenced on the DNBSEQ platform using paired-end 100 bp reads (BGI Genomics, China).

Queen Mary’s Apocrita HPC facility^43^ was used to process sequenced raw data. Read quality was assessed using FastQC^44^ (version 0.12.1), and summary reports were generated using MultiQC^45^ (version 1.28). Adapter sequences and low-quality bases were removed using Trimmomatic^46^ (version 0.39; SLIDINGWINDOW:4:20; MINLEN:20; 3′ bases with mean Phred < 20 were trimmed and reads < 20 bp discarded). Post-trimming quality was re-evaluated using FastQC v0.12.1, with results summarized using MultiQC v1.29. Trimmed reads were aligned to the *Rattus norvegicus* reference genome (mRatBN7.2, Ensembl release 113) using HISAT2^47^ (version 2.2.1) with default parameters. SAMtools^48^ (version 1.19.2) was used to convert, sort, and index alignment files. An average alignment rate of >99% was achieved across all samples. Gene-level read quantification was performed using featureCounts^49^ (Subread v2.0.1), with approximately 83% of reads assigned to annotated gene features (Ensembl release 113). Quality control analysis using principal component analysis (PCA) and sample-to-sample heatmaps with hierarchical clustering identified samples CKD_5A and SOTA_4A as outliers and were excluded from downstream analyses. Differential expression analysis was conducted in RStudio (R version 4.4.2) using the DESeq2^50^ package (version 1.46.0). *P*-values were adjusted for multiple correction testing using Benjamini-Hochberg method and genes with padj < 0.05 were identified to be significantly differentially expressed.

### 2.12 Cardionet *in silico* modelling

*In silico* simulations were performed using the metabolic network of the cardiomyocyte CardioNet.^9^ Mathematical modelling has previously been used to study the dynamics of cardiac metabolism in response to stress and CardioNet has been successfully applied to identify limiting metabolic processes and estimate flux distributions.^9^ Flux balance analysis allows to estimate flux rates in a cellular model based on metabolic constraints that are defined by the extracellular environment (e.g. oxygen and nutrient supply), cellular demands (e.g. proliferation, contraction) and tissue type. This modelling approach combines biochemical network models with optimality problems, which describe different cost or benefit functions and allow us to include experimental data for example metabolite levels, enzyme levels or flux rates.

Optimization problems were defined with the objective to minimize the total sum of fluxes through the CardioNet metabolic network.^51^ Simulations for sham, CKD or CKD SOTA-treated groups were based on the assumption that cardiomyocytes seek to maintain a certain ATP provision to sustain cardiac contractile function alongside the synthesis of macromolecules, including structural proteins and phospholipids for membranes.^51^ We included the intracellular metabolite concentrations of 51 metabolites: acetyl-CoA, α-glucose, citrate, citrulline, CoA, cytosine, fumarate, glucose-6-phosphate, glutathione, glycine, alanine, arginine, asparagine, aspartate, cystathionine, glutamate, glutamine, isoleucine, leucine, lysine, malate, methionine, phenylalanine, threonine, ornithine, α-ketoglutarate, phosphoenolpyruvate, pyruvate, glycogen, acetyl carnitine, choline, phosphocholine, carnitine, S-Adenosylhomocysteine, S-Adenosylmethionine, serine, succinate, aconitate, NAD, NADH, NADP, NADPH, ADP, AMP, ATP, tyrosine, taurine, creatine, phosphocreatine, valine, and acetate. Furthermore, we constrained extracellular metabolites that could be taken up from the blood, including glucose, lactate, cholesterol and free fatty acids. Linear programming was solved using the GUROBI solver (version 9.1.2 build v9.1.2rc0, Linux64).^52^

Z-scores were calculated to visualize how many standard deviations an estimated flux rate is away from the mean across all experimental groups. The z-score describes the distance from the mean for a given flux rate as a function of the standard deviation. For example, a z-score equal to 1 represents a flux for a given experimental group that is 1 standard deviation greater than the mean across all experimental groups.

### 2.13 *In silico* modelling of E-C coupling

We investigated the effects of elevated Na_i_ on cardiomyocyte Ca^2+^ dynamics using the modified Leeds model of rat ventricular myocyte electrophysiology.^53^ This model was selected for its improved action potential duration (APD) rate dependence and ionic current magnitudes consistent with experimental data. For both control and elevated sodium conditions, cells were paced at a cycle length of 250 ms for 300 beats before analysis.

### 2.14 Statistical analysis

Data are presented as mean ± SEM. Normality of data distribution was examined using Shapiro–Wilk’s normality test. Comparisons between multiple groups were performed by one-way ANOVA with Holm–Šídák post-hoc test or Kruskal–Wallis test with Dunn’s post-hoc test for non-normally distributed data. For cardiac metabolomic data, values were log2-transformed and expressed as fold change relative to the appropriate group mean for each comparison. Statistical significance of fold changes was assessed using a one-sample *t*-test against zero. Statistical analysis was performed using GraphPad Prism (v10.2.2) software. Differences were considered significant when *P* < 0.05.

## 3. Results

### 3.1 CKD induces early cardiac phenotype characterized by intracellular Na^+^ overload

Four weeks following partial nephrectomy (*Figure 1A*), CKD animals develop renal dysfunction (*Figure 1B, C*), anaemia (*Figure 1D*), and hypertension.^3,29–34^ At this early stage, cardiac structure remained preserved with no evidence of LVH (*Figure 1E*, *Table 1*). While systolic function (ejection fraction, *Figure 1F*) was maintained, reduced E/A ratio (*Figure 1G*) together with prolonged mitral valve deceleration time (∼60% increase; *Table 1*) was consistent with early diastolic dysfunction. Body weights (see Supplementary material online, *Figure S1*) as well as plasma metabolic and liver function markers (*Table 2*) were comparable between CKD and sham animals. At the early stage of 4-week CKD, the metabolomic profiles of liver, muscle, and kidney did not change (see Supplementary material online, *Figure S2A–C*).

**Figure 1:**
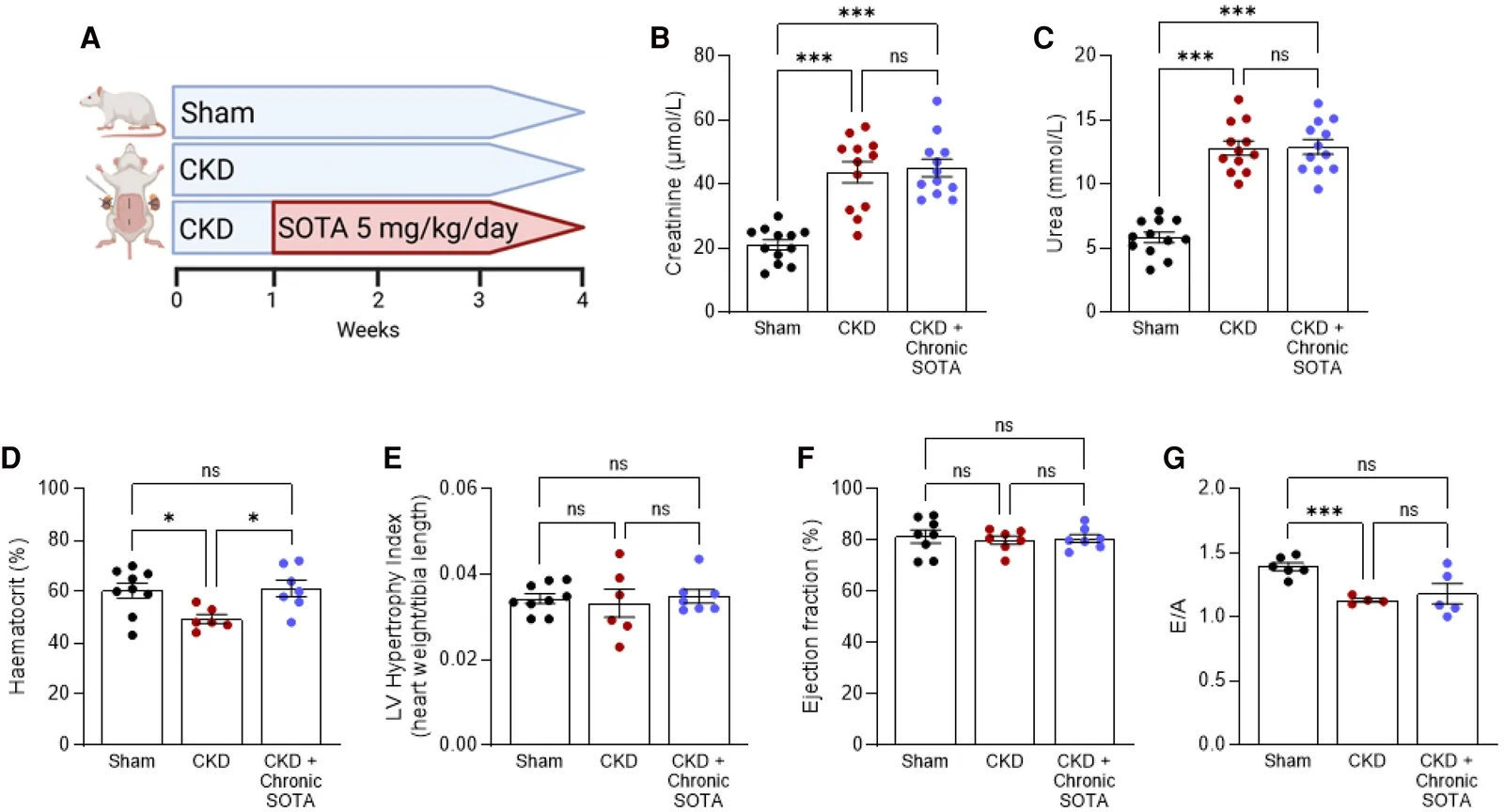
CKD phenotype 4 weeks post-PN induction. (*A*) Schematic summary of the CKD induction protocol (created with BioRender.com), (*B*, *C*) Plasma levels of creatinine and urea, (*D*) Haematocrit, (*E*) left ventricle hypertrophy as a ratio of heart weight/tibia length, (*F*, *G*) Echocardiography measurements of ejection fraction and E/A ratio. Analysed by one-way ANOVA, Holm-Šídák post-hoc displayed, *P < 0.05, ***P < 0.001. *n* numbers: (*A–B*) Sham *n* = 12, CKD *n* = 12, CKD + Chronic SOTA *n* = 12; (*C–D*) Sham *n* = 9, CKD *n* = 6, CKD + Chronic SOTA *n* = 7; (*F*) Sham *n* = 9, CKD *n* = 7, CKD + Chronic SOTA *n* = 7; (*G*) Sham *n* = 6, CKD *n* = 4, CKD + Chronic SOTA *n* = 5.

**Table 1:** Echocardiography assessment of 4-week CKD

	Sham	CKD	CKD + Chronic SOTA
Fractional shortening (%)	50.67 ± 2.93	50.33 ± 1.59	51.40 ± 1.78
Mitral valve deceleration time (ms)	24.10 ± 3.14	38.60 ± 5.33	33.76 ± 4.49
Intraventricular septum, end-diastole (mm)	1.98 ± 0.05	1.86 ± 0.11	1.89 ± 0.09
Intraventricular septum, end-systole (mm)	3.29 ± 0.13	3.21 ± 0.17	3.35 ± 0.12
Left ventricle internal diameter, end-diastole (mm)	7.28 ± 0.35	6.91 ± 0.31	7.92 ± 0.19
Left ventricle internal diameter, end-systole (mm)	3.60 ± 0.30	3.42 ± 0.19	3.79 ± 0.12
Left ventricular posterior wall, end-diastole (mm)	2.23 ± 0.09	2.28 ± 0.12	2.04 ± 0.12
Left ventricular posterior wall, end-systole (mm)	3.46 ± 0.14	3.45 ± 0.15	3.43 ± 0.15
End systolic volume (µL)	56.90 ± 11.42	50.12 ± 6.65	65.85 ± 4.94
End diastolic volume (µL)	287.8 ± 32.3	250.2 ± 24.6	343.4 ± 17.2

Data displayed as mean ± SEM. Analysed by one-way ANOVA with Holm-Šídák post-hoc test (Sham *n* = 9, CKD *n* = 7, CKD + Chronic SOTA *n* = 7), except mitral valve deceleration time which was analysed by Kruskal-Wallis test with Dunn’s post-hoc test (Sham *n* = 6, CKD *n* = 4, CKD + Chronic SOTA *n* = 5).

**Table 2:** Plasma analysis of 4-week CKD

	Sham	CKD	CKD + Chronic SOTA
Glucose (mmol/L)	10.98 ± 0.47	10.07 ± 0.42	10.61 ± 0.26
Triglycerides (mmol/L)	1.44 ± 0.23	1.46 ± 0.14	1.32 ± 0.19
Free fatty acids (µmol/L)	397.6 ± 44.94	553.2 ± 76.02	355.7 ± 30.94^#^
Alanine transaminase (U/L)	36.75 ± 3.39	38.17 ± 3.42	52.92 ± 5.11*^#^
Alkaline phosphatase (U/L)	106.6 ± 8.95	120.8 ± 8.02	141.2 ± 14.46

Data displayed as mean ± SEM. Analysed by one-way ANOVA with Holm-Šídák post-hoc test, except alanine transaminase which was analysed by Kruskal-Wallis test with Dunn’s post-hoc test. *P < 0.05 vs. Sham, #*P* < 0.05 vs. CKD. Sham *n* = 12 (FFAs, triglycerides *n* = 11), CKD *n* = 12 (FFAs *n* = 11), CKD + Chronic SOTA *n* = 12.

Despite modest structural and functional changes at this early stage of CKD, ^23^Na NMR spectroscopy of Langendorff-perfused CKD hearts (*Figure 2A*) revealed a significant increase in Naᵢ (*Figure 2B*), identifying Naᵢ overload as an early feature of CKD-driven cardiac remodelling.

**Figure 2:**
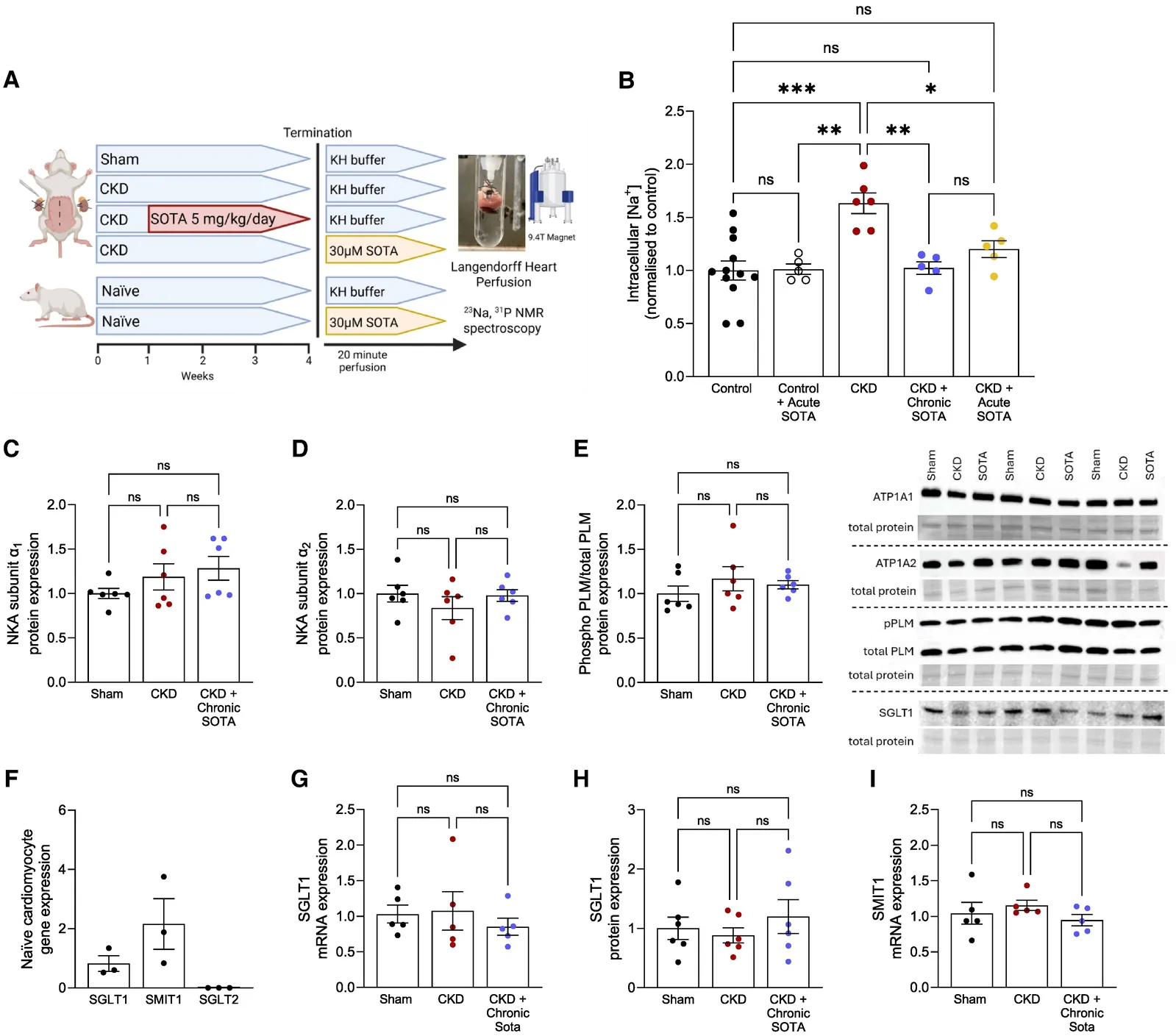
Myocardial Na_i_ assessment in CKD hearts. (*A*) Schematic summary of experimental design (created with BioRender.com). Na_i_ measured in CKD hearts following chronic (in vivo, 3 weeks) or acute (20 min, ex vivo perfusion). A separate cohort of naïve hearts were subjected to acute SOTA treatment. (*B*) Na_i_ assessed by triple quantum filtered ^23^Na spectroscopy in Langendorff perfused heart. [Na^+^] shown relative to control. Baseline intracellular [Na^+^] for Sham and Naïve hearts were pooled and are presented as a single control group. Analysed by one-way ANOVA, Holm-Šídák post-hoc displayed; *P < 0.05, **P < 0.01, ***P < 0.001; Control *n* = 12, Control + acute SOTA *n* = 5, CKD *n* = 6, CKD + Chronic SOTA *n* = 5, CKD + Acute SOTA *n* = 5. Protein expression of (*C*) Na/K-ATPase (NKA) subunit α1, (*D*) NKA subunit α2, and (*E*) phosphorylated (S68) phospholemman (PLM) in cardiac tissue. NKA subunit expression normalized to total protein. Phosphorylated PLM expressed relative to total PLM. Data shown in C-E Analysed by one-way ANOVA, Holm-Šídák post-hoc displayed, n = 6 for all groups. (*F*) Gene expression of SGLT1, SMIT1 and SGLT2 in isolated cardiomyocytes from naïve Wistar rats displayed relative to housekeeping gene 36B4, *n* = 3. (*G*) Gene expression of SGLT1 in cardiac tissue displayed relative to housekeeping gene 36B4, *n* = 5 for all groups. (*H*) Protein expression of SGLT1 in cardiac tissue expressed relative to total protein, *n* = 6 for all groups. (*I*) Gene expression of SMIT1 in cardiac tissue displayed relative to housekeeping gene 36B4, *n* = 5 for all groups. Data shown in G-I analysed by one-way ANOVA, Holm-Šídák post-hoc test.

### 3.2 Na_i_ overload is associated with *in silico* predicted Ca^2+^ dysregulation independent of transcriptional changes

RNA sequencing revealed no significant changes in the expression of Na_i_ or Ca^2+^ handling genes (see Supplementary material online, *Table S2*), indicating that observed ionic dysregulation arises from functional alterations rather than transcriptional remodelling. To determine the functional consequences of elevated Naᵢ, we performed *in silico* modelling of rat cardiomyocyte electrophysiology incorporating myocardial Naᵢ levels measured by ^23^Na NMR spectroscopy (*Figure 2B*). Simulations showed that elevating cardiomyocyte Naᵢ to the levels observed in CKD hearts impaired Ca^2+^ handling (*Figure 3*), producing abnormalities similar to those reported in other models of LVH and HF, in which disrupted Ca^2+^ homeostasis is a recognized feature.^5,8,54^ Specifically, a 67% increase in Naᵢ, as measured in CKD hearts (*Figure 2B*) led to increased Na^+^/K^+^ pump current (INaK, *Figure 3A*), reduced fast sodium current (INa, *Figure 3B*), and weaker forward-mode Na^+^/Ca^2+^ exchanger current (INCX, *Figure 3C*). These changes contributed to a shorter APD and a lower action potential peak in the simulations (*Figure 3D*). The reduced peak voltage resulted in a larger L-type calcium current (ICaL) amplitude (*Figure 3E*). Combined with diminished Ca^2+^ extrusion via NCX (*Figure 3C*), this led to elevated systolic and diastolic Ca^2+^ (*Figure 3F*), consistent with diastolic dysfunction observed in CKD *in vivo* (*Figure 1G*).

**Figure 3:**
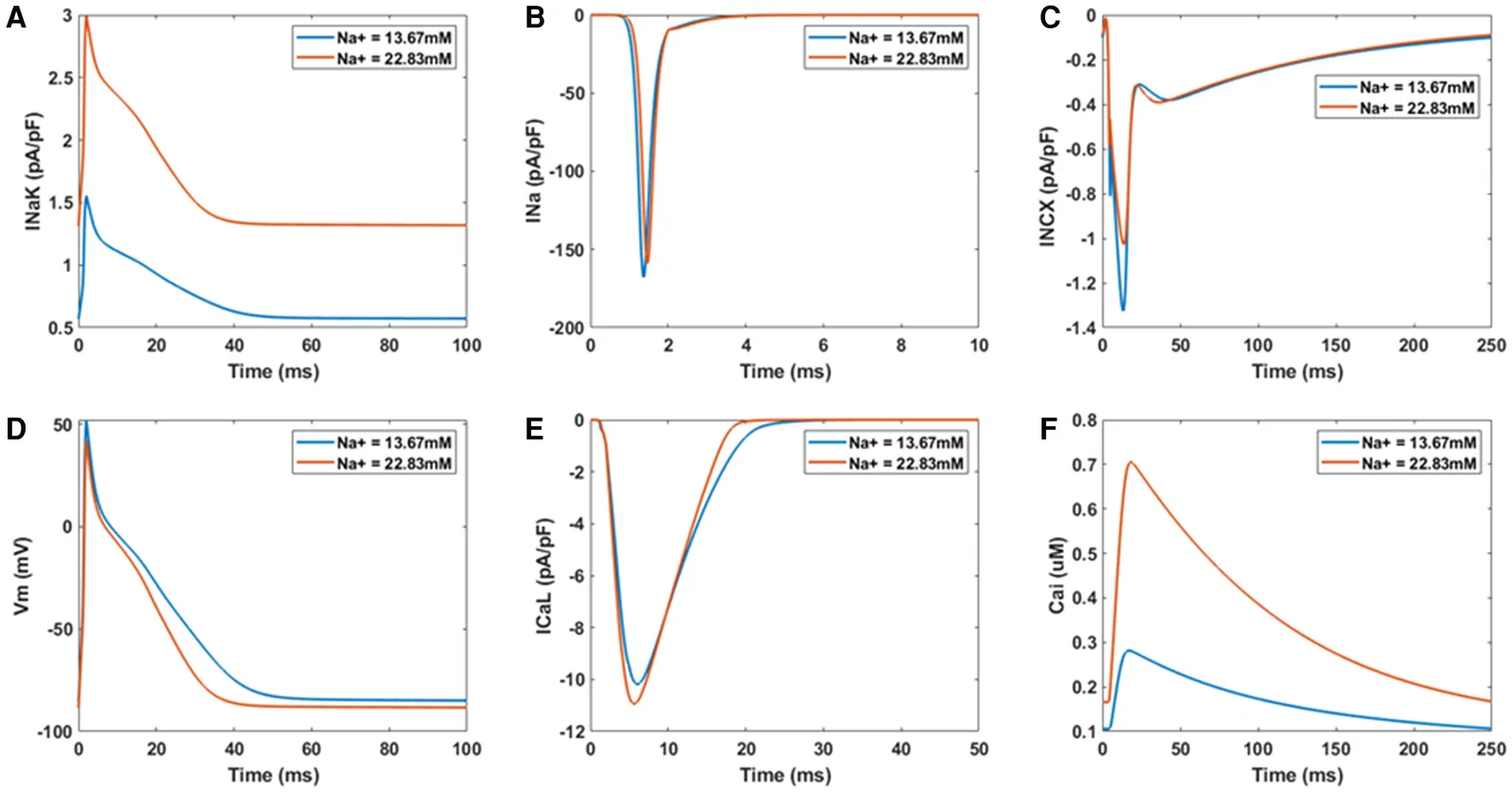
*In silico* predicted consequences of elevated myocardial Na_i_^+^ on key sodium and calcium currents, action potential, and calcium dynamics in a rat cardiomyocyte. To mimic changes we observed in CKD, intracellular Na^+^ was increased by 67% (from 13.67 mM to 22.83 mM) in a model of rat cardiomyocyte. Compared with control, the elevated Na model exhibited: (*A*) increased Na^+^/K^+^ pump current (INaK), (*B*) reduced fast sodium current (INa), and (*C*) weaker forward-mode Na^+^/Ca^2+^ exchanger current (INCX). These changes contributed to a shorter APD and a lower action potential peak (*D*). The reduced peak voltage resulted in a larger L-type calcium current (ICaL) amplitude (*E*). Combined with diminished Ca^2+^ extrusion via NCX (*C*), this led to elevated systolic and diastolic Ca^2+^ (*F*).

### 3.3 Na_i_ overload is associated with cardiac metabolic reprogramming in CKD

Cardiac metabolomic profiling demonstrated a distinct metabolic signature in CKD hearts. PCA revealed a clear separation between the CKD and sham group, driven predominantly by PC1 (82.5% of the total variance) (*Figure 4A*), indicating substantial metabolic remodelling. GLUT4 mRNA expression was reduced (*Figure 4B*), suggesting impaired glucose uptake, whilst CD36 expression was unchanged (see Supplementary material online, *Figure S3*). Metabolomic analysis (LC-MS/MS and ^1^H NMR) revealed a shift in redox and substrate metabolism, with an increased NADP/NADPH ratio and accumulation of glycogen, citrulline, aspartate, methionine and cystathionine, whilst acetyl-CoA, acetyl carnitine, choline and glutamine levels were reduced (*Figure 4C*). These changes are consistent with disruption of mitochondrial substrate utilization, activation of one-carbon and trans-sulfuration pathways, and altered nitrogen metabolism. Despite distinct metabolic divergence, myocardial energetic status was preserved, with no difference in PCr/ATP between groups (*Figure 4D*).

**Figure 4:**
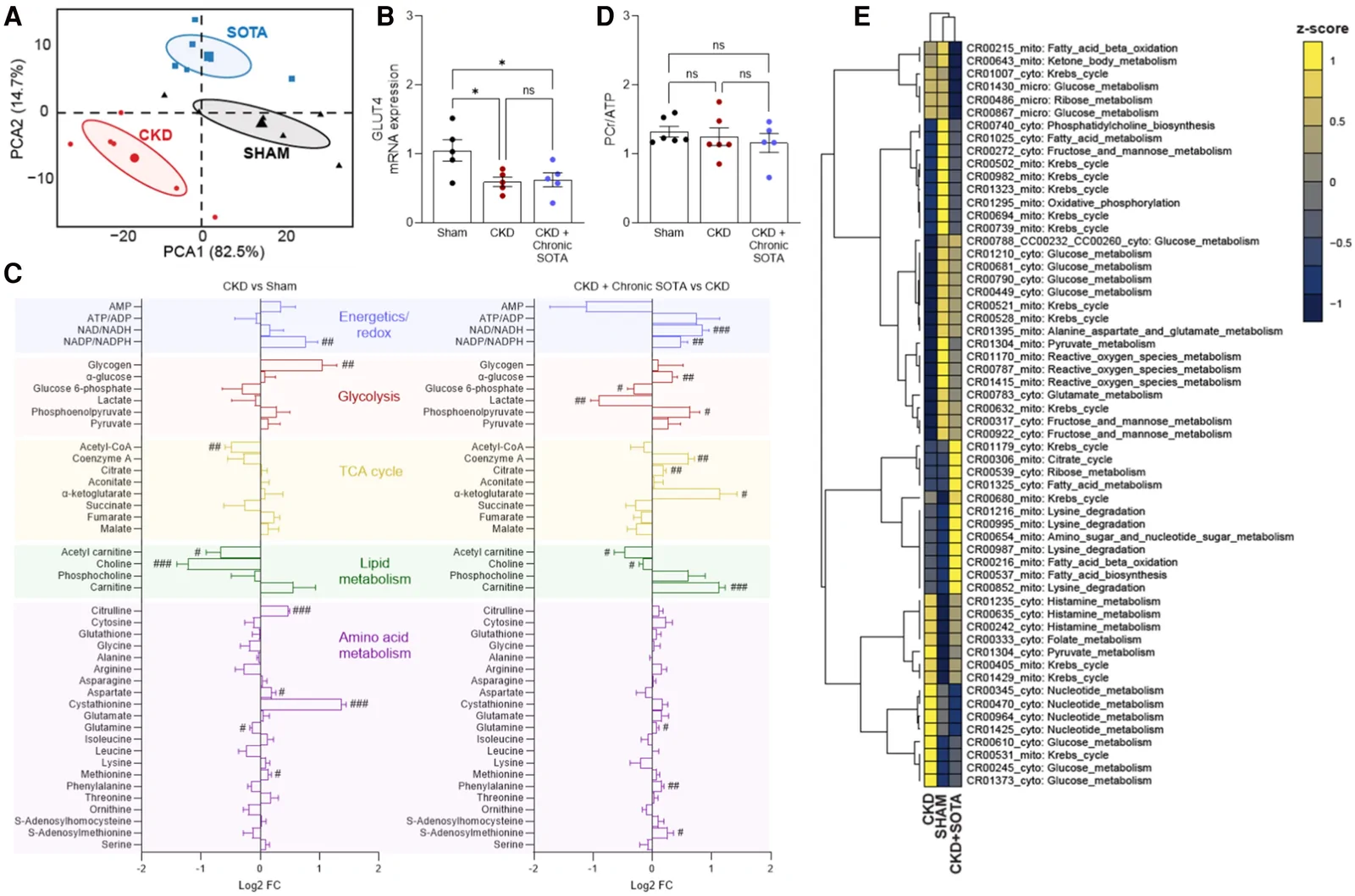
Cardiac metabolic profile of 4-week CKD following chronic SOTA treatment. (*A*) Principal component analysis of ^1^H NMR spectroscopy and LC-MS/MS metabolomic profile. (*B*) Gene expression of GLUT4 in cardiac tissue displayed relative to housekeeping gene 36B4. Analysed by one-way ANOVA and Holm-Šídák post-hoc test, *P < 0.05, *n* = 5 for all groups. (*C*) Cardiac metabolomic profile; metabolite abundances were log2 transformed and expressed as fold change relative to corresponding control group for each comparison. Analysed by one-sample *t*-test against zero, ^#^*P* < 0.05, ^##^*P* < 0.01, ^###^*P* < 0.001. (*D*) *In situ* perfused heart ^31^P NMR spectroscopy assessment of PCr/ATP. Analysed by one-way ANOVA, Holm-Šídák post-hoc test, *P < 0.05, Sham *n* = 6, CKD *n* = 6, CKD + Chronic SOTA *n* = 5. (*E*) Unsupervised hierarchical clustering of estimated z-scored flux rate changes reveals metabolic adaptation in response to CKD development and SOTA treatment. Heat maps summarize results for reactions in the Krebs cycle, OXPHOS, and glucose metabolism. Flux distributions were calculated by flux balance analysis using the mammalian network of cardiac metabolism, CardioNet. The colour scale indicates the degree to which estimated flux rate changes are predicted to be respectively lower or higher. CardioNet modelling based on cardiac LC-MS/MS (*n* = 6 per group), cardiac ^1^H NMR (*n* = 7 per group) and plasma analysis (Sham *n* = 8, CKD *n* = 9, CKD + Chronic SOTA *n* = 5).

Flux-based modelling (*Figure 4E*) further demonstrated widespread suppression of metabolic activity in CKD hearts with reduced flux through Krebs cycle, glycolysis, fatty acid metabolism and oxidative phosphorylation (blue heatmap Z-score, *Figure 4E*). This was accompanied by reduced predicted O_2_ utilization (see Supplement Supplementary material online, *Figure S4*), indicative of impaired mitochondrial oxidative function. Cardionet network-level visualization confirmed a global reduction in pathway flux intensity in CKD hearts, reflected by markedly diminished reaction edge thickness across the metabolic network (*Figure 5*).

**Figure 5:**
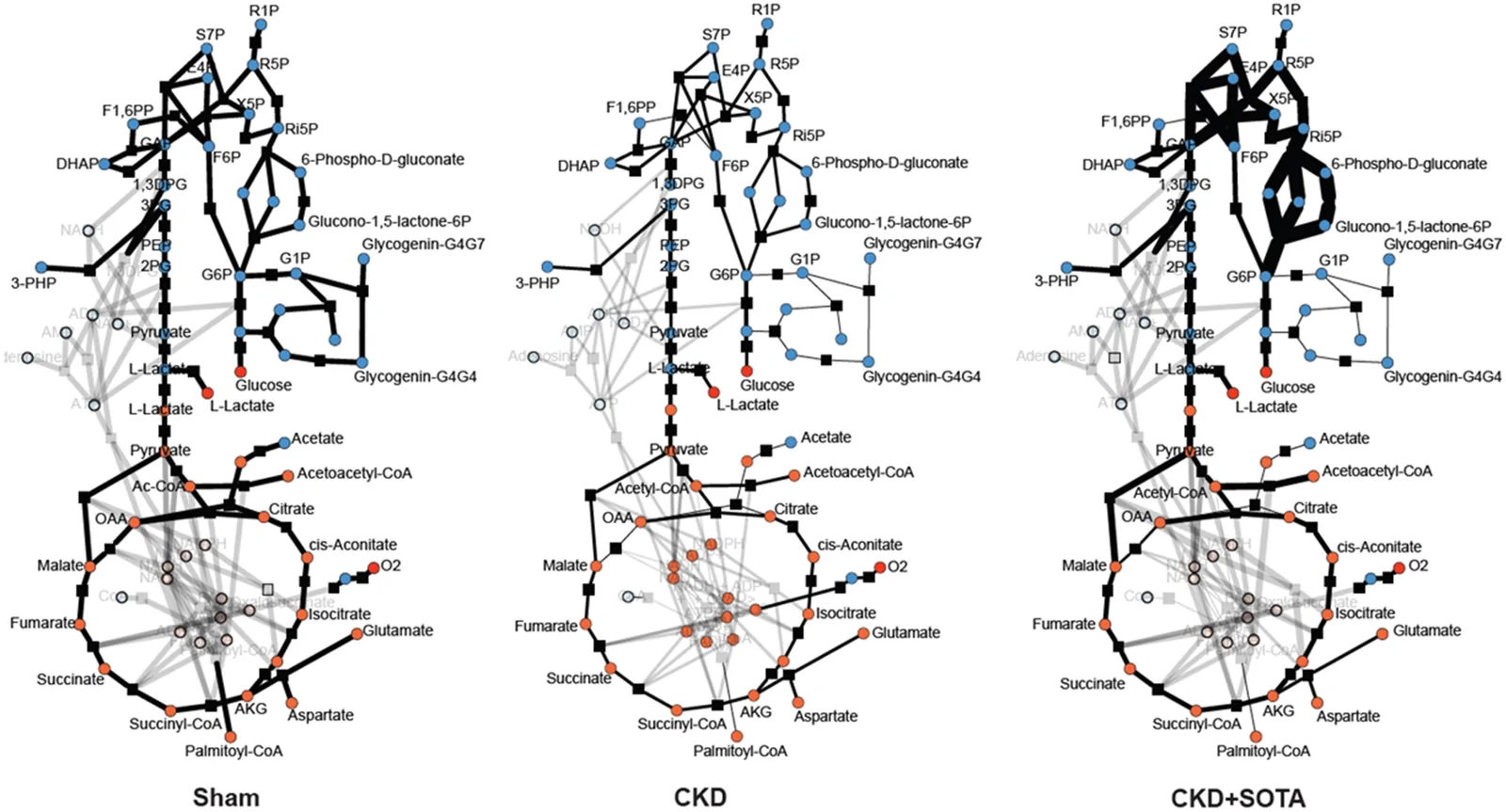
CardioNet *in silico* metabolic flux changes in response to CKD and chronic SOTA treatment. Coloured nodes represent metabolites assigned to five different compartments: extracellular space, cytosol, mitochondria, microsome, lysosome. The black square nodes indicate reactions; two reactions are linked by a directed edge indicating the reaction flux. The line thickness of each edge is proportional to predicted flux rate change. CardioNet modelling based on cardiac LC-MS/MS (*n* = 6 per group), cardiac ^1^H NMR (*n* = 7 per group) and plasma analysis (Sham *n* = 8, CKD *n* = 9, CKD + Chronic SOTA *n* = 5).

### 3.4 SOTA normalizes Na_i_ in CKD myocardium

Following the observation of elevated Naᵢ in CKD myocardium, we next examined its potential as a therapeutic target. Chronic SOTA administration (3 weeks, 5 mg/kg/day, protocol summary *Figure 2A*) normalized elevated Naᵢ in CKD hearts (CKD vs. Control, *P* < 0.001; CKD vs. CKD + Chronic SOTA, *P* < 0.01; *Figure 2B*).

To establish whether this effect was cardiac-specific, CKD hearts were acutely perfused with SOTA *ex vivo*. Acute treatment (30 µM, 20 min, *Figure 2A*) reduced Naᵢ to a comparable extent as chronic *in vivo* SOTA administration, supporting a direct myocardial effect (*Figure 2B*, acute SOTA 27% reduction vs. CKD *P* < 0.05, Chronic SOTA 37% reduction vs. CKD *P* < 0.01). In contrast, acute SOTA administration had no effect on myocardial Naᵢ in healthy control hearts (*Figure 2B*, Control 0.67 ± 0.02, Control + acute SOTA 0.67 ± 0.03, *P* = 0.997).

This Naᵢ normalization occurred independently of transcriptional regulation of canonical Na handling pathways as the protein expression of Na^+^/K ^+^ -ATPase (α_1_, α_2_ subunits) phospholemman (PLM, total and phosphorylated Ser68) and mRNA level of Na^+^/H^+^ exchangers (NHE1, NHE3) was unchanged by CKD or SOTA treatment (*Figure 2C–E*, Supplementary material online, *Table S2*). Analysis of isolated cardiomyocytes showed that SGLT1 is expressed in cardiac cells, whereas SGLT2 is absent (*Figure 2F*). The expression of SGLT1, at both the protein and RNA levels, remained unchanged (*Figure 2G, H*) as did that of its regulatory protein, SMIT1 (*Figure 2I*).

### 3.5 Na_i_ normalization improves cardiac metabolic profile in CKD

SOTA-induced normalization of Naᵢ was accompanied by marked remodelling of the cardiac metabolome. PCA demonstrated clear separation of SOTA-treated CKD hearts from untreated CKD along PC2 (14.7% variance), indicating partial reversal of the disease-associated metabolic phenotype (*Figure 4A*). While PCr/ATP ratio remained unchanged (*Figure 4D*), SOTA treatment improved redox balance, with increased NAD^+^/NADH ratio (*Figure 4C*). Metabolite profiling revealed increased glucose, phosphoenolpyruvate and carnitine alongside markedly reduced lactate and glucose-6-phosphate compared to untreated-CKD, consistent with reduced reliance on anaerobic glycolysis (*Figure 4C*). Krebs cycle intermediates citrate and α-ketoglutarate were increased, accompanied by increased coenzyme A (*Figure 4C*), indicating altered mitochondrial substrate utilization. There were mild changes in the amino acid metabolism compared to non-treated CKD with increase of glutamine, phenylalanine and S-adensylmethionine (*Figure 4C*).

Flux modelling (*Figure 4E*) demonstrated that SOTA reverses many CKD-induced changes in the heart as many pathways in heatmap analysis show decreased activity in CKD (blue colour) and increased activity in SOTA + CKD (yellow colour, *Figure 4E*). This pattern is especially strong in the Krebs cycle, many mitochondrial pathways, glucose, fatty acid, and pyruvate metabolism (*Figure 4E*). Thus, SOTA treatment appears to restore central carbon metabolism and improve mitochondrial function. Moreover, network analysis (*Figure 5*) demonstrated a densely interconnected cluster of glycolytic and pentose phosphate intermediates, consistent with coordinated remodelling of central carbon metabolism following SOTA treatment. Collectively, these findings demonstrate that normalization of Naᵢ is associated with improved redox state, reduced glycolytic dependence, and improvement in mitochondrial oxidative function in the heart.

Systemically, CKD was associated with a 42% increase in circulating fatty acids compared with controls, although this did not reach statistical significance (*Table 2*). However, SOTA treatment significantly reduced fatty acid levels, restoring them to values comparable to sham controls (*Table 2*). Alanine transaminase levels were significantly higher in the SOTA-treated group compared with both control and CKD groups (*Table 2*). CKD showed no significant systemic metabolic differences compared with control at baseline. SOTA induced tissue-specific metabolic changes, increasing acetyl carnitine, glutamate, acetate, and succinate in liver while reducing alanine, NADH, ATP, glycine, and aspartate (see Supplementary material online, *Figure S2D*). In muscle, acetate and fumarate were increased and succinate and glycine reduced (see Supplementary material online, *Figure S2E*). In the remnant kidney, α-glucose and creatinine were elevated, whereas myoinositol, GTP, carnitine, and glutamate were decreased (see Supplementary material online, *Figure S2F*).

### 3.6 Na_i_-driven metabolic remodelling is reflected in the cardiac transcriptome

To determine whether Naᵢ-associated metabolic remodelling is reflected at the transcriptional level, we performed bulk RNA sequencing. Principal component analysis (*Figure 6A*) demonstrated partial separation between CKD and sham groups, with CKD samples displaying greater dispersion.

**Figure 6:**
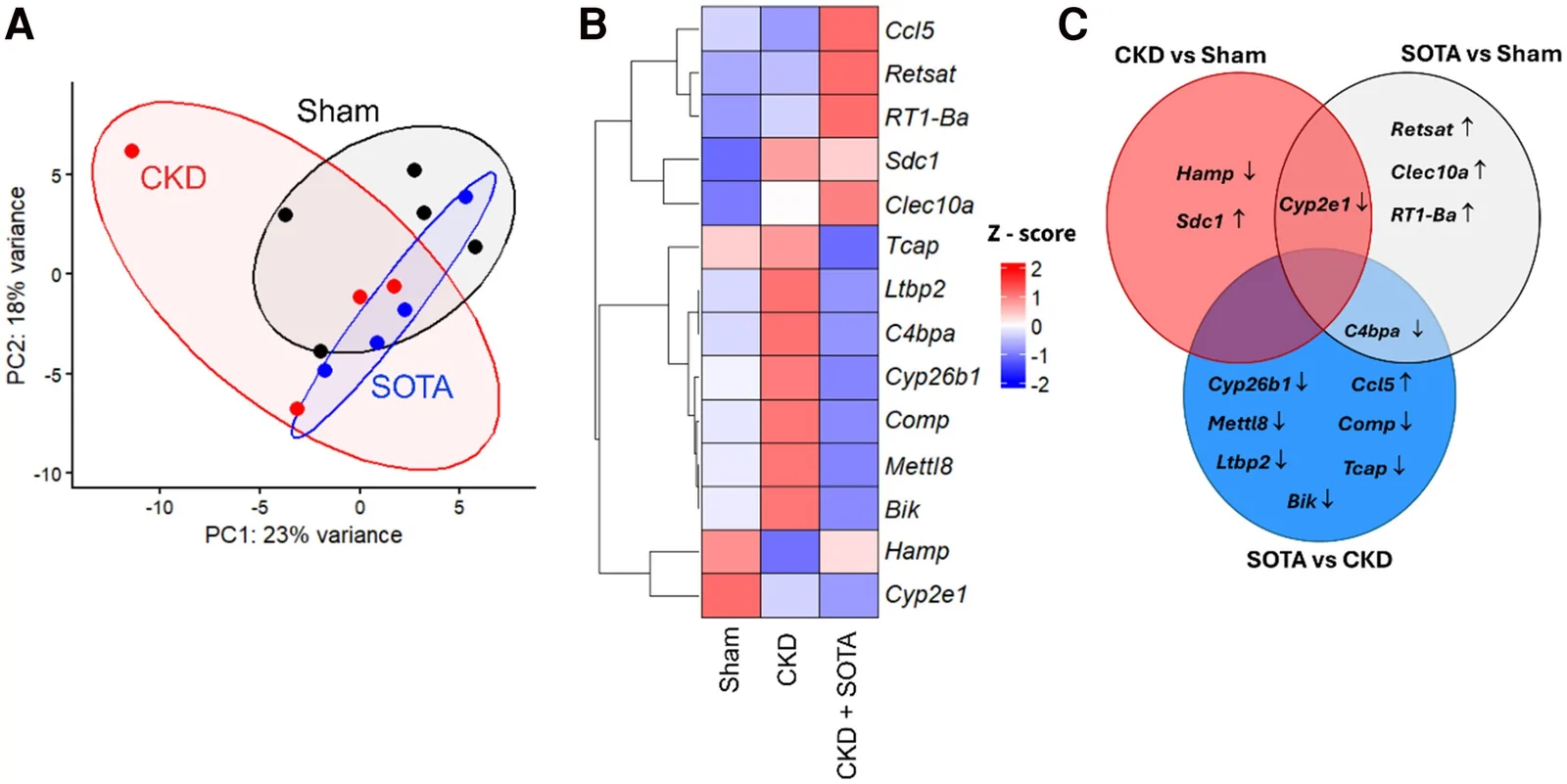
Cardiac transcriptomic profile of 4-week CKD following chronic SOTA treatment. (*A*) Principal component analysis identified three distinct cardiac transcriptomic profiles. (*B*) Heatmap of differentially expressed genes (DEGs). (*C*) Venn diagram showing overlap of DEGs. DEGs filtered by |log_2_FC| ≥ 0.5 and *P*adj < 0.05). Upregulated and downregulated genes are indicated by arrows. Groups: Sham *n* = 5, CKD *n* = 4, CKD + SOTA *n* = 4.

SOTA-treated CKD samples formed a distinct cluster, separating from CKD and shifting towards the sham control group, indicating a treatment-associated modulation of the transcriptional profile.

Compared with sham, CKD induced modest transcriptional changes in the heart (*Figure 6B*), characterized by increased *Sdc1* expression and reduced *Hamp* and *Cyp2e1* levels (*Figure 6C*). SOTA treatment elicited a distinct gene expression profile (*Figure 6B*), with upregulation of *Retsat, Clec10a*, and *RT1-Ba* compared with sham (*Figure 6C*). Relative to CKD, SOTA treatment downregulated a subset of genes associated with extracellular matrix remodelling and inflammation, including *Comp*, *Ltbp2*, *Ccl5*, and *Bi*k, as well as *Cyp26b1* (*Figure 6C*), indicating targeted modulation of disease-associated pathways.

### 3.7 SOTA enhances mitochondria-nucleus communication

CKD was associated with reduced cardiac expression of mitonuclear tethering proteins TSPO and TMPO, suggesting impaired mitochondria-nucleus communication (*Figure 7*). In CKD hearts, SOTA treatment stabilized TSPO expression and partially restored TMPO isoforms, with TMPOγ showing the most marked restoration. These findings indicate improved cellular stress signalling and mitochondrial-nuclear coordination (*Figure 7*).

**Figure 7:**
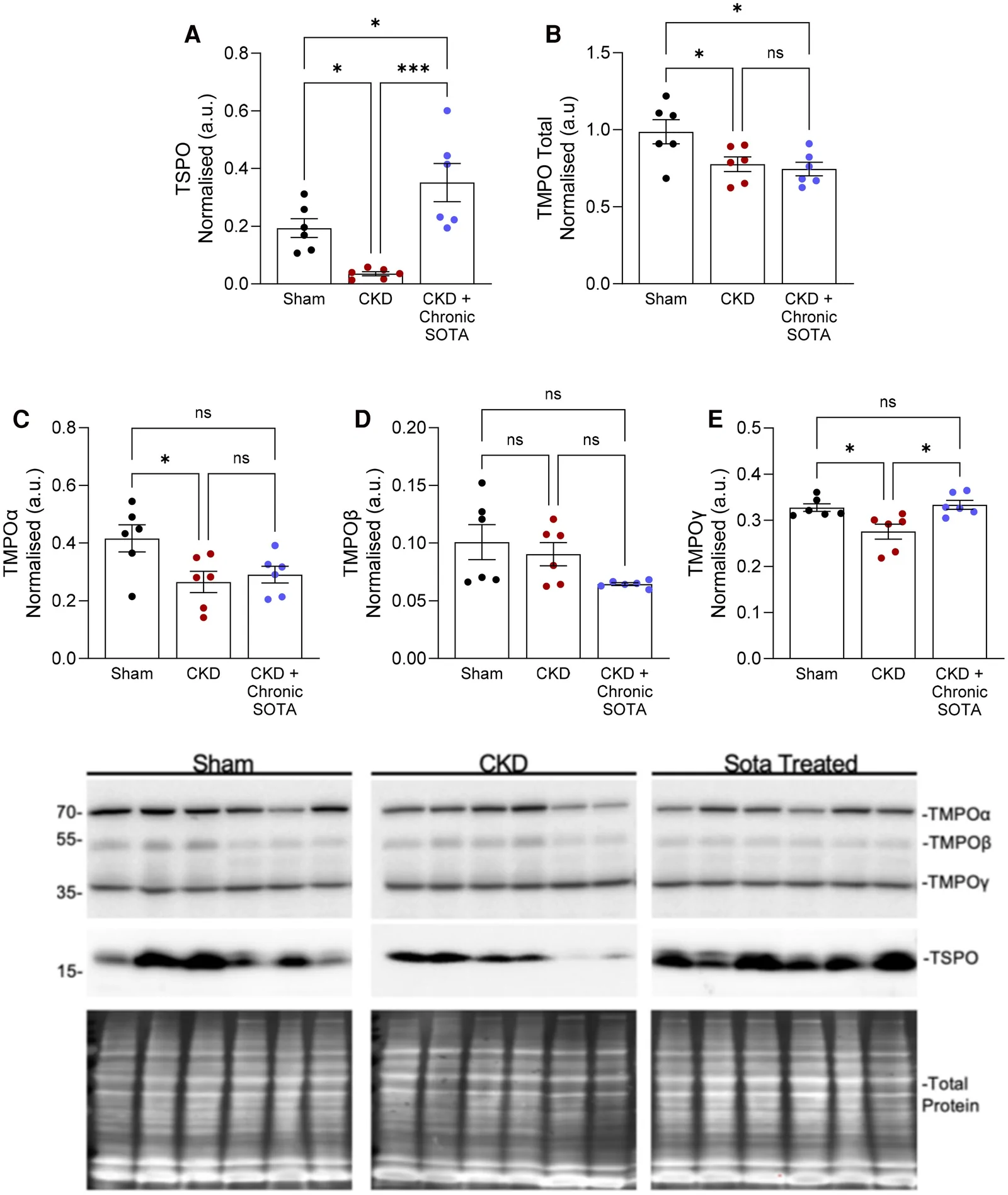
SOTA treatment improves myocardial mitochondria-nucleus contact. Western blot analysis of cardiac tissue from CKD hearts following SOTA treatment showed changes in the protein level of key mediators of mitochondria-nucleus contact. Protein level of (*A*) TSPO, (*B*) total TMPO, and three TMPO isoforms: (*C*) TMPOα, (*D*) TMPOβ and (*E*) TMPOγ. Protein level normalized to total protein. Analysed by one-way ANOVA with Holm-Šídák post-hoc test, *P < 0.05, ***P < 0.001, *n* = 6 for all groups.

## 4. Discussion

In this study, we identify Naᵢ accumulation as an early, therapeutically modifiable feature of uraemic cardiomyopathy. At 4 weeks post-partial nephrectomy, CKD hearts exhibited elevated myocardial Naᵢ preceding LVH and reduced ejection fraction, but with established diastolic dysfunction. Naᵢ elevation was associated with a distinct metabolic signature, including suppression of central carbon metabolism, redox imbalance, and in silico-predicted impairment of mitochondrial oxidative flux, while high-energy phosphate reserve was preserved. These findings position Naᵢ dysregulation upstream of structural remodelling and indicate that early uraemic cardiomyopathy reflects metabolic inefficiency rather than energetic failure observed later in CKD.^35^

Increased cardiomyocyte Naᵢ is a recognized feature of failing myocardium and is closely linked to abnormal Ca^2+^ homeostasis through effects on the Na^+^/Ca^2+^ exchanger and related ion transport pathways.^9,55–58^ In keeping with this framework, electrophysiological simulations using the experimentally measured elevated Naᵢ values predicted reduced forward-mode NCX activity, altered action potential morphology, and elevation of Ca^2+^. The absence of detectable changes in transcripts encoding major Na^+^- and Ca^2+^-handling proteins alongside the simulation results suggest that Na_i_ dysregulation arises predominantly from functional rather than transcriptional remodelling. The predicted Ca^2+^ phenotype agrees with the diastolic impairment observed *in vivo* and provides a plausible mechanistic link between CKD, Naᵢ overload, and early contractile dysfunction.

The underlying cause of elevated Na_i_ in CKD has long been debated, with earlier hypotheses implicating either reduced efflux due to altered NKA expression or function, or increased influx through the activation of background sodium channels.^2,56,59–64^ Additionally, CKD is associated with elevated levels of circulating NKA inhibitors, such as cardiotonic steroids and uraemic toxins, which are thought to contribute to chronic Na_i_ overload.^2^ The rapid SOTA-driven fall in Naᵢ in isolated perfused CKD hearts argues against SOTA effect being explained solely by changes in glycaemia, renal haemodynamics, or circulating uraemic factors. Equally important, acute SOTA had no effect on [Naᵢ] in healthy control hearts, indicating a disease-selective action on pathological Na^+^ handling leading to accumulation.

The metabolic phenotype of CKD hearts was consistent with known consequences of Naᵢ elevation.^8,9,55^ Cardiac metabolomics and flux modelling demonstrated reduced glycolytic, tricarboxylic acid cycle, fatty acid, and oxidative phosphorylation fluxes, together with lower predicted oxygen utilization, despite preservation of PCr/ATP. This pattern suggests a myocardium that remains energetically compensated at rest but operates with reduced oxidative flexibility. Overall, these observations align with our previous work showing that Na^+^ elevation can directly reprogramme cardiac metabolism before overt energetic and functional failure develops.^9^

A major finding of the present study is that SOTA normalized cardiac Naᵢ after both chronic treatment *in vivo* and acute administration *ex vivo*, supporting a direct myocardial action. SGLT inhibitors have been shown to lower myocardial Na_i_^+^ and modulate Ca^2+^ handling through SGLT2-independent mechanisms. Uthman *et al*.^27^ demonstrated that dual SGLT1/2 inhibition with canagliflozin reduced Na^+^  _i_ and inhibited NHE-1 activity in isolated mouse ventricular cardiomyocytes, while Hamouda *et al*.^65^ reported that dapagliflozin reduced systolic intracellular Ca^2+^ in STZ-diabetic rats but not in controls. Consistent with these observations and given the absence of cardiac SGLT2 (*Figure 2F*), we show that SOTA rapidly lowers Na_i_ in Langendorff-perfused hearts, supporting a direct cardiac effect that may involve SGLT1 inhibition and/or non-canonical Na^+^-handling pathways.^66^

What molecular mechanisms underlie the observed increase in myocardial Naᵢ in CKD? Given changes in NKA expression or PLM phosphorylation were not observed, we hypothesize that enhanced Na_i_ influx *via* SGLT1 could become an important contributor to Naᵢ accumulation in CKD (hypothesis summary *Figure 8*). SGLT1 is expressed in cardiomyocytes, and its Na-coupled glucose transport activity has been shown to be upregulated in cardiac disease.^67^ Previous work by Lambert *et al*.^21^ in HF identified a contribution of SGLT1 to cytosolic Naᵢ overload, particularly in the presence of systemic metabolic derangements such as obesity and diabetes. Moreover, humans with decreased functional SGLT1 were shown to exhibit improved survival and decreased prevalence of HF.^68^ Although SGLT1 transcript levels were unchanged, altered transporter activity may drive pathological Naᵢ influx in CKD. Consistent with this, recent work implicates cardiac SGLT1 in metabolic and functional remodelling, suggesting activity, not expression, governs cardiomyocyte ionic and metabolic homeostasis.^69^ Although we hypothesize that enhanced SGLT1 activity may contribute to increased myocardial Naᵢ in CKD, SGLT1 activity was not directly measured in this study and should be investigated in future work.

**Figure 8:**
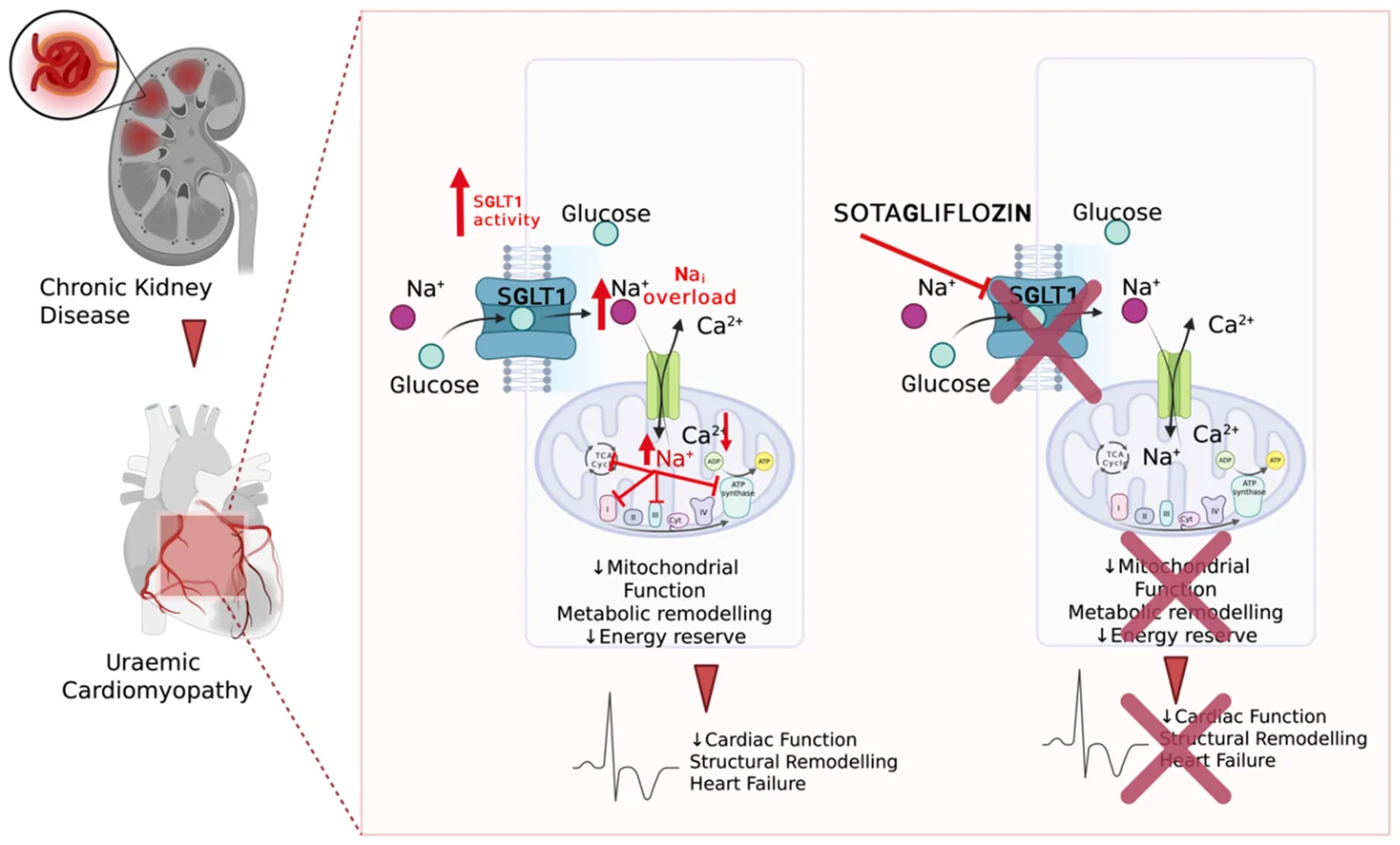
Proposed mechanism of Na_i_ overload in CKD cardiomyopathy and impact of sotagliflozin. Created with BioRender.com.

Naᵢ normalization with SOTA improved the cardiac metabolic phenotype, shifting the myocardial metabolome away from CKD: redox balance improved and central carbon flux was restored. Network analysis showed greater connectivity in glycolytic and pentose phosphate pathways, with reversal of CKD-associated suppression of mitochondrial metabolism (pyruvate, Krebs cycle, fatty acid pathways).

These changes occurred without altering PCr/ATP, indicating improved intermediary metabolism rather than energetic reserve, supported by increased myocardial carnitine consistent with enhanced fatty acid metabolism.

The systemic metabolome contextualizes these cardiac findings. Early CKD caused minimal systemic disturbance, whereas SOTA induced tissue-specific changes in liver, skeletal muscle, and the remnant kidney, alongside reduced circulating fatty acids and improved anaemia. Enhanced O_2_ delivery may support mitochondrial function and ion homeostasis, but the acute *ex vivo* Naᵢ-lowering effect indicates this is not solely explained by anaemia correction. Given emerging heart-liver metabolic crosstalk, SOTA may also promote interorgan metabolic signalling.^70,71^

Transcriptomic profiling supports early activation of disease-relevant myocardial pathways in CKD and suggests that SOTA modifies this response. Although overall gene expression changes were modest at this early CKD stage, hearts showed a transcriptional signature consistent with fibrotic signalling, metabolic adaptation, and disrupted redox and iron homeostasis. Upregulation of *Sdc1*, a mediator of glycocalyx integrity and cardiac fibrosis,^72,73^ together with downregulation of *Cyp2e1*, linked to oxidative stress and mitochondrial dysfunction,^74,75^ suggests early extracellular matrix and redox remodelling. In parallel, reduced *Hamp* expression raises the possibility of altered cardiomyocyte iron handling, a process critical for mitochondrial function and regulated by the hepcidin-ferroportin axis, although this remains hypothesis-generating in the absence of direct measurements of myocardial iron content or transporter activity.^76–78^ SOTA induced a distinct transcriptional signature characterized by suppression of genes associated with extracellular matrix remodelling, inflammation, and apoptosis, including *Comp, Ltbp2, Ccl5*, and *Bik*, consistent with attenuation of maladaptive remodelling pathways. In addition, coordinated regulation of retinoic acid-related genes, with downregulation of *Cyp26b1* and upregulation of *Retsat*, may indicate preservation of retinoid signalling, which has been implicated in limiting LVH and fibrosis.^79–81^ Nevertheless, these findings should be interpreted cautiously given the modest scale of differential expression and the absence of direct functional validation but are directionally consistent with the metabolic and functional phenotype observed.

CKD hearts displayed reduced expression of TSPO and TMPO, suggesting disruption of mitochondria-nucleus communication and mitonuclear signalling.^82–84^ This interface is essential for coordinated expression of nuclear-encoded mitochondrial proteins, retrograde signalling, and adaptive responses to metabolic stress.^85^ When disrupted, mitonuclear communication can impair mitochondrial biogenesis, compromise oxidative phosphorylation, increase ROS production, and exacerbate pathological remodelling, arrhythmias, and cardiomyocyte apoptosis.^82–84^ TSPO, an outer mitochondrial membrane protein, mediates the formation of nuclear-mitochondrial contact sites (NAM) through interactions with nuclear binding partners, while TMPO contributes to stabilizing these inter-organellar contacts.^82–84^ In CKD, dysregulated expression of TSPO and TMPO compromises the physical proximity required for adaptive signalling. SOTA partially restored the expression of both TSPO and TMPO, particularly the nuclear-envelope-associated TMPOγ isoform, suggesting a potential role in re-establishing NAM and improving mitonuclear communication. By repositioning mitochondria adjacent to the nucleus, SOTA may facilitate stabilization of transcription factors and promote expression of stress-adaptive genes, thereby supporting metabolic and redox homeostasis under CKD conditions. These observations implicate a mitochondria-centric mechanism whereby SOTA integrates cellular stress sensing with nuclear transcriptional adaptation. While the precise molecular mechanisms remain to be fully elucidated, these findings provide supportive evidence that restoration of mitonuclear signalling may be an important component of SOTA’s cardioprotective effects. Future studies will explore the functional consequences of restored TSPO and TMPOγ signalling, including mitochondrial dynamics, nuclear signalling, and cardiomyocyte function.

As a dual SGLT1/2 inhibitor, SOTA may also exert effects *via* SGLT2-specific inhibition. SGLT2-specific inhibitors have been proposed to lower Naᵢ via off-target modulation of Na^+^ transporters, including the Na^+^/H^+^ exchanger (NHE1), with downstream improvement in redox balance and reduced oxidative stress.^86,87^ However, we found no evidence of altered NHE1/NHE3 transcript levels; furthermore, as shown in our electrophysiological simulations, the elevation of Naᵢ seen in CKD would naturally diminish the inward driving force for Na_i_ through such transporters. Consequently, any off-target blockade of NHE1 by SOTA would likely serve to further interrupt this Na_i_ loading, thereby mitigating the downstream Ca^2+^ overload and electrical remodelling illustrated in *Figure 3*. Additional class effects may involve modulation of O-GlcNAcylation and downstream phosphorylation of CaMKII, late Na^+^ current (I_na_,L), and excitation-contraction coupling proteins (see Supplementary material online, *Figure S5*).^7,88,89^ However, given the absence of systemic hyperglycaemia and the rapid Naᵢ reduction observed in our ex vivo experiments, acute O-GlcNAc-dependent mechanisms are unlikely to fully explain the effect. SGLT inhibition may also exert cardioprotective effects independent of Na^+^ handling, including modulation of endothelial nitric oxide signalling.^90^ We acknowledge that selective SGLT1-targeted experiments represent important future work to directly establish causality and definitively delineate the relative contributions of SGLT1 vs. off-target effects.

Question remains how these mechanistic findings translate clinically. Although our data implicate SGLT1 inhibition in regulating myocardial Naᵢ, large trials so far show cardiovascular benefit with selective SGLT2 inhibitors.^17,18^ This likely reflects convergence on shared downstream pathways rather than dependence on transporter selectivity alone.^22,26,27^ Furthermore, clinical outcomes reflect integrated systemic responses, including renal haemodynamics, metabolic adaptation, and neurohormonal signalling.^17,18^

Translational perspective Myocardial sodium overload emerges as an early and potentially reversible feature of uraemic cardiomyopathy, preceding overt systolic dysfunction and hypertrophy. This study identifies intracellular Na^+^ accumulation as a mechanistically relevant driver of cardiometabolic abnormalities in chronic kidney disease (CKD), providing a new framework linking renal dysfunction to cardiac remodelling. Importantly, dual SGLT1/2 inhibition with sotagliflozin rapidly normalized myocardial Na^+^ and improved cardiac metabolic homeostasis, including in isolated perfused hearts, supporting a direct myocardial effect. These findings suggest that targeting myocardial Na^+^ dysregulation may represent a novel therapeutic strategy for preventing or attenuating CKD-associated heart failure, particularly HFpEF.

## 5. Study limitations

Although our findings support a direct cardiac effect of dual SGLT1/2 inhibition on Na_i_ handling, we did not directly assess SGLT1 transporter activity or glucose uptake in isolated cardiomyocytes. These experiments fall outside the scope of the present study and represent important avenues for future work. We have recently demonstrated that prolonged CKD progression (12 weeks) in PN model results in severe cardiac dysfunction and impaired ischaemic stress tolerance.^35^ Our study focuses on early CKD, before overt LVH or systolic dysfunction, and therefore does not establish whether Naᵢ lowering modifies progression to advanced CKD cardiomyopathy. Finally, metabolomic flux predictions and transcriptomic associations provide system-level insight but do not replace direct flux measurements or functional testing of the implicated pathways.

### 5.1 Conclusion

This study identifies myocardial Naᵢ overload as an early hallmark of CKD cardiomyopathy and shows that dual SGLT1/2 inhibition with SOTA rapidly normalizes Naᵢ and is associated with improved metabolic homeostasis. These findings support the concept that disturbed cardiac Na^+^ handling is not merely a consequence of advanced heart failure, but an early and potentially targetable driver of CKD-driven cardiac remodelling.

## Supplementary Material

cvag167_Supplementary_Data

## Data Availability

All underlying data supporting the findings of this study are provided in the Excel file submitted alongside the manuscript. RNA-sequencing data generated in this study is available open access on Array Express under accession **E-MTAB-1604.**
