# Supplementary material for: Dual inhibition of SGLT 1 and 2 by sotagliflozin alleviates intracellular sodium overload and improves cardiometabolic remodelling in uraemic cardiomyopathy
Source: Cardiovasc Res. 2026 Aug 11;122(14):2039–54. doi: 10.1093/cvr/cvag167 (PMC13653498; doi:10.1093/cvr/cvag167)
Supplement: Supplementary file 1 [file cvag167_supplementary_data.zip › Supplementary material - SOTA rebuttal_FINAL.docx]

**Supplementary Material**

**Supplementary Tables:**

**Supplementary Table 1. Primer sequences**

| Primer | Forward Primer Sequence | Reverse Primer Sequence |
| --- | --- | --- |
| 36B4 | AGAGGTGCTGGACATCACAG | CATTGCGGACACCCTCTAG |
| Glut 4 | AGAGGAGAGGGCGGACATT | ACTCTTCATTCAGGCCCTTGG |
| CD36 | GCTGATTACTTCTGTGTAGTAGCTT | TCCTCGTGCAGCAGAATCAA |
| SGLT1 | CAAAATCGCCTGTGTCCTCC | AGGCCTCGAAGTCCGTTAGG |
| SGLT2 | ATTGGTGTTGGCTTGTGGTC | AGCGCATTCCACTCAAATCC |
| SMIT1 | CCAGCCTCGGTATGGTACTG | GCATCTCCACAATAATTGGC |

**Supplementary Table 2. Differential expression analysis of sodium and calcium handling genes.** Differential expression was assessed using DESeq2. Log2 fold changes (log2FC) are shown for the comparisons along with p-values adjusted for multiple testing using the Benjamini–Hochberg false discovery rate method (padj).

| Gene | Encoded protein | Comparison | Log2FC | padj |
| --- | --- | --- | --- | --- |
| *SLC8A1* | NCX1 | CKD vs. Sham | -0.034535902 | 0.979330426 |
|  |  | CKD vs CKD + Chronic SOTA | -0.060902913 | 0.995618293 |
|  |  | CKD + Chronic SOTA vs. Sham | 0.02636701 | 0.999840801 |
| *ATP2A2* | SERCA2 | CKD vs. Sham | -0.034418409 | 0.963605727 |
|  |  | CKD vs CKD + Chronic SOTA | -0.019004305 | 0.996612502 |
|  |  | CKD + Chronic SOTA vs. Sham | -0.015414104 | 0.999840801 |
| *ATP1A1* | Na⁺/K⁺ ATPase α1 | CKD vs. Sham | -0.124189383 | 0.90808327 |
|  |  | CKD vs CKD + Chronic SOTA | -0.144703956 | 0.949904255 |
|  |  | CKD + Chronic SOTA vs. Sham | 0.020514573 | 0.999840801 |
| *SLC8B1* | NCLX | CKD vs. Sham | 0.038722007 | 0.981192477 |
|  |  | CKD vs CKD + Chronic SOTA | 0.039502224 | 0.996612502 |
|  |  | CKD + Chronic SOTA vs. Sham | -0.000780217 | 0.999840801 |
| *SLC9A1* | NHE1 | CKD vs. Sham | -0.118724527 | 0.90808327 |
|  |  | CKD vs CKD + Chronic SOTA | -0.224125058 | 0.557041185 |
|  |  | CKD + Chronic SOTA vs. Sham | 0.105400531 | 0.999840801 |
| *SLC9A3* | NHE3 | CKD vs. Sham | 0.734325175 | 0.90808327 |
|  |  | CKD vs CKD + Chronic SOTA | 0.213168635 | 0.995618293 |
|  |  | CKD + Chronic SOTA vs. Sham | 0.521156539 | 0.999840801 |
| *CACNA1C* | Cav1.2 | CKD vs. Sham | 0.05165038 | 0.96987237 |
|  |  | CKD vs CKD + Chronic SOTA | 0.109634863 | 0.995618293 |
|  |  | CKD + Chronic SOTA vs. Sham | -0.057984483 | 0.999840801 |
| *CACNB2* | Cavβ2 | CKD vs. Sham | -0.115125556 | 0.932470414 |
|  |  | CKD vs CKD + Chronic SOTA | 0.092509185 | 0.995618293 |
|  |  | CKD + Chronic SOTA vs. Sham | -0.207634741 | 0.999840801 |
| *SLC12A2* | NKCC | CKD vs. Sham | 0.107910192 | 0.926894205 |
|  |  | CKD vs CKD + Chronic SOTA | 0.166314188 | 0.973071639 |
|  |  | CKD + Chronic SOTA vs. Sham | -0.058403996 | 0.999840801 |

**Supplementary Figure 1. Body weight of animals following PN induction of CKD.** (A) Change in body weight over time following two stage PN induction of CKD and (B) terminal body weight expressed as percentage of initial weight. Analysed by one-way ANOVA, Holm-Šídák post-hoc displayed. Sham n=14, CKD n=18, CKD + Chronic SOTA n=12

**Supplementary Figure 2. Metabolomic profile of peripheral tissues in CKD and following chronic sotagliflozin treatment.** ^1^H NMR analysis of liver, muscle and kidney tissue in CKD animals (A-C) and CKD animals chronically treated with sotagliflozin (D-F). Each point represents the fold change (FC) of a metabolite plotted against the associated level of statistical significance for the change analysed by t-test (horizontal dashed line indicates p=0.05 threshold). All tissues: n=5 per group.

**Supplementary Figure 3.** Gene expression of CD36 in cardiac tissue displayed relative to housekeeping gene 36B4. Analysed by one-way ANOVA, Holm-Šídák post-hoc displayed, n=5 for all groups.

**Supplementary Figure 4.** CardioNet *in silico* cardiac metabolic modelling of flux rates for amino acids (threonine, lysine, isoleucine) and oxygen. CardioNet modelling based on cardiac LC-MS/MS (n=6 per group), cardiac ^1^H NMR (n=7 per group) and plasma analysis (Sham n=8, CKD n=9, CKD + Chronic SOTA n=5).

**Supplementary Figure 5. Potential [Na^+^]_i_ lowering effect of SOTA by O-GlcNAcylation‐dependent mechanisms as a class-effect independent of SGLT1/2 selectivity**
